# Protocol: mixed-methods study of how implementation of US state medical cannabis laws affects treatment of chronic non-cancer pain and adverse opioid outcomes

**DOI:** 10.1186/s13012-020-01071-2

**Published:** 2021-01-07

**Authors:** Emma E. McGinty, Kayla N. Tormohlen, Colleen L. Barry, Mark C. Bicket, Lainie Rutkow, Elizabeth A. Stuart

**Affiliations:** 1 Baltimore, USA; 2 Ann Arbor, USA

**Keywords:** Cannabis, Law, Mixed-methods, Policy implementation

## Abstract

**Background:**

Thirty-three US states and Washington, D.C., have enacted medical cannabis laws allowing patients with chronic non-cancer pain to use cannabis, when recommended by a physician, to manage their condition. However, clinical guidelines do not recommend cannabis for treatment of chronic non-cancer pain due to limited and mixed evidence of effectiveness. How state medical cannabis laws affect delivery of evidence-based treatment for chronic non-cancer pain is unclear. These laws could lead to substitution of cannabis in place of clinical guideline-discordant opioid prescribing, reducing risk of opioid use disorder and overdose. Conversely, state medical cannabis laws could lead to substitution of cannabis in place of guideline-concordant treatments such as topical analgesics or physical therapy. This protocol describes a mixed-methods study examining the implementation and effects of state medical cannabis laws on treatment of chronic non-cancer pain. A key contribution of the study is the examination of how variation in state medical cannabis laws’ policy implementation rules affects receipt of chronic non-cancer pain treatments.

**Methods:**

The study uses a concurrent-embedded design. The primary quantitative component of the study employs a difference-in-differences design using a policy trial emulation approach. Quantitative analyses will evaluate state medical cannabis laws’ effects on treatment for chronic non-cancer pain as well as on receipt of treatment for opioid use disorder, opioid overdose, cannabis use disorder, and cannabis poisoning among people with chronic non-cancer pain. Secondary qualitative and survey methods will be used to characterize implementation of state medical cannabis laws through interviews with state leaders and representative surveys of physicians who treat, and patients who experience, chronic non-cancer pain in states with medical cannabis laws.

**Discussion:**

This study will examine the effects of medical cannabis laws on patients’ receipt of guideline-concordant non-opioid, non-cannabis treatments for chronic non-cancer pain and generate new evidence on the effects of state medical cannabis laws on adverse opioid outcomes. Results will inform the dynamic policy environment in which numerous states consider, enact, and/or amend medical cannabis laws each year.

Contributions to the literature

- The majority of policy implementation research focuses on strategies for enacting evidence-based policy. This protocol describes methods for studying how implementation of enacted public policy influences policy effects on health outcomes.
- The study described in this protocol uses quantitative measures of key implementation outcomes—acceptability, adoption, appropriateness, and penetration—in a public policy evaluation context.
- The study described in this protocol describes a strategy for unpacking the “black box” of variation in implementation of a single type of policy (in this case, medical cannabis laws) across multiple states. We use moderation analyses within a difference-in-differences framework to study whether specific policy implementation rules (i.e., law provisions and regulations) moderate laws’ effects on outcomes.

## Background

Chronic non-cancer pain, defined as pain stemming from conditions other than cancer that occurs on at least half of days for 6 months or more [1], affects 20% of US adults aged 18+ and 28% of adults aged 65+ [2]. Cannabis is a potentially effective treatment for chronic non-cancer pain, but evidence is limited and subject to varying interpretations. For example, a 2017 National Academies of Science, Engineering and Medicine (NASEM) report concluded that cannabis is an effective treatment for chronic non-cancer pain in adults [3], while a 2018 Cochrane review identified no high-quality studies and concluded that the risks of cannabis for chronic non-cancer pain may outweigh the benefits [4]. While patients with chronic non-cancer pain are eligible to use cannabis for pain management under all extant US state medical cannabis laws, which are in place in 33 US states and D.C., no clinical guidelines currently recommend cannabis for chronic non-cancer pain.

Since 2016, Centers for Disease Control and Prevention clinical guidelines have recommended non-opioid, non-cannabis treatments such as topical analgesics and physical therapy as the first-line treatments for chronic non-cancer pain [5]. While opioids were a clinically accepted first-line treatment for chronic non-cancer pain from the late 1990s to mid-2010s, current guidelines indicate that the risks of opioid treatment often outweigh the benefits [5]. Opioid prescribing for chronic non-cancer pain has played a significant role in the US opioid overdose crisis, which was driven in part by opioid overprescribing [6]. One study concluded that adults with arthritis, a common chronic pain condition, made up more than half of all adults taking prescribed opioids in 2013 [7].

Some prior studies suggest that state medical cannabis laws may be associated with reductions in opioid prescribing, opioid use disorder, and opioid overdose [8–16], while others suggest that medical cannabis laws may lead to increased nonmedical opioid use and overdose [17, 18]. The available research is limited by five key factors, which the study described in this protocol is designed to overcome:

1. Failure to consider variation in the implementation of state medical cannabis laws

While the majority of policy implementation research has focused on implementation strategies for and barriers and facilitators to enacting evidence-based policy [19–26], studying the implementation of enacted public policies is critically important: the degree to which an enacted policy is implemented determines whether and how that policy will affect outcomes. In this study, we focus upon two primary elements of policy implementation: (1) policy implementation rules and (2) policy implementation outcomes.

We define policy implementation rules as statutory provisions or regulations delineating how the policy will be implemented. In the medical cannabis context, examples include rules allowing or disallowing sale of dry-leaf cannabis, the cheapest form, which may increase patient access but also increase risk of diversion to nonmedical use; rules specifying the allowable volume and location of dispensaries; and rules that “medicalize” medical cannabis programs (i.e., align with standard medical practice), for example state rules requiring physicians to undergo specialized training in order to recommend medical cannabis and rules limiting patients to a 30-day supply of medical cannabis [27, 28]. Prior studies examining medical cannabis laws’ effects on opioid-related outcomes have not accounted for variation in policy implementation rules, which occurs both across and within states over time as state policymakers enact and amend medical cannabis laws. However, research examining state cannabis laws’ effects on other outcomes, such as diversion of medical cannabis to non-medical use, suggests that variation in policy implementation rules contributes to heterogeneous policy effects on outcomes [29–31].

Implementation outcomes are well defined in the implementation science literature [32], but are rarely measured in the context of policy implementation; in particular, a recent systematic review identified a dearth of quantitative implementation outcome measures in policy studies [33]. Our study uses qualitative research to measure Proctor et al.’s eight implementation outcomes [32] (acceptability, appropriateness, adoption, costs, feasibility, fidelity, penetration, and sustainability) and survey research to measure four implementation outcomes: acceptability, adoption, appropriateness, and penetration. These implementation outcomes are critical to interpreting econometric policy evaluation results; for example, if implementation measures show robust substitution of medical cannabis in place of prescription opioids to treat chronic non-cancer among healthcare providers and patients, this finding strengthens confidence in (hypothetical) econometric policy evaluation findings suggesting that medical cannabis laws were associated with decreases in prescription opioid use.

(2) Lack of triangulation of quantitative econometric analysis results with findings, generated from other data collection methods, on implementation outcomes

No national or state data sources track individual-level medical cannabis use alongside chronic non-cancer pain diagnoses and treatments. In the absence of such data, insurance claims data can be used to examine prescription opioid and other pain treatment use in a longitudinal cohort of chronic pain patients over time; however, cannabis is not covered by insurers and therefore not measurable in these data [34, 35]. Thus, studies cannot observe patient-level substitution of cannabis in place of prescription opioids or non-opioid treatments (even if such data existed, substitution is not always observable, i.e., when a physician recommends cannabis in a scenario where they would—in the absence of a medical cannabis law—have recommended opioids to a patient not currently using opioids). Given this limitation, the ability to make causal inferences from quantitative policy evaluations is strengthened by triangulation with findings, front other data collection methods, on policy implementation. Large effects of medical cannabis laws on opioid-related outcomes are not plausible in the absence of high acceptability, appropriateness, adoption, and penetration of medical cannabis to manage chronic pain among clinicians treating and patients experiencing chronic non-cancer pain. As noted above, our study uses both qualitative (interview) and quantitative (survey) methods to measure policy implementation alongside a rigorous difference-in-differences quantitative policy evaluation.

(3) Lack of consideration of important non-opioid outcomes

No studies have examined how state medical cannabis laws influence clinical guideline-concordant treatment for chronic non-cancer pain or how these laws affect cannabis use disorder and cannabis poisoning among people with chronic non-cancer pain; this study considers these outcomes.

(4) Lack of individual-level longitudinal cohort studies

This study examines the effects of state medical cannabis laws on opioid prescribing in a longitudinal cohort of individuals over time, in contrast to prior studies using aggregate, state-level cross-sectional data.

(5) General population samples

Studies associating medical cannabis laws with improved opioid outcomes have explained their results as due to substitution of cannabis in place of opioids for chronic non-cancer pain [10–16]. But, these studies have used general population samples, which could bias results as people without chronic non-cancer pain in the sample are not expected to be affected by medical cannabis laws but are likely affected by other state laws put in place at or around the same time. State opioid prescribing laws including prescription drug monitoring program (PDMP), pill mill, and acute pain opioid prescribing cap laws—widely adopted in the early-to-mid 2010s [36, 37]—do not target patients with chronic non-cancer pain, but have been shown to affect receipt of opioid prescriptions in other segments of the US population [38–48]. Studies using general population samples are vulnerable to policy endogeneity, or inability to disentangle the effects of state medical cannabis and opioid prescribing laws. The quantitative policy evaluation study described in this protocol uses a longitudinal cohort of adults with chronic non-cancer pain diagnoses.

## Methods

### Study aims and hypotheses

#### Aim 1

Study aim 1 is to examine the effects of state medical cannabis laws on receipt of clinical guideline-discordant opioid and clinical guideline-concordant non-opioid, non-cannabis treatment among patients with chronic non-cancer pain. We will use difference-in-differences design with a policy trial emulation approach [49] adapted from comparative effectiveness research to identify the comparison group. We expect state medical cannabis laws to reduce receipt of opioid and non-opioid, non-cannabis treatment among patients with low back pain, headache, fibromyalgia, arthritis, and/or neuropathic pain.

#### Aim 2

Study aim 2 is to examine the effects of state medical cannabis laws on receipt of treatment for opioid use disorder, opioid overdose, cannabis use disorder, and cannabis poisoning among patients with chronic non-cancer pain. We will use the same difference-in-differences design with policy trial emulation approach as in aim 1. We expect state medical cannabis laws to decrease utilization for opioid use disorder and opioid overdose and to increase utilization of treatment for cannabis use disorder and cannabis poisoning among patients with low back pain, headache, fibromyalgia, arthritis, and/or neuropathic pain.

#### Aim 1–2 hypotheses related to policy implementation rules

In aims 1–2, we will analyze how specific policy implementation rules modify laws’ effects on outcomes. Hypotheses pertaining to specific policy implementation rules are shown in Table 1.

**Table 1:** Aim 1–2 hypotheses related to state cannabis law implementation rules

***Hypotheses related to specific state medical cannabis policy implementation rules (aims 1–2)*** 1. *Medicalization*: Relative to less medicalized laws, laws with a higher degree of medicalization—shown to decrease medical cannabis program enrollment—will have a lesser effect on aim 1–2 outcomes. 2. *Non-specific chronic pain provisions*: Laws that include broad “non-specific” chronic pain qualifying criteria will have greater effects on outcomes relative to laws with narrower criteria, e.g., a requirement of a headache specifically. 3. *Dry-leaf provisions*: Laws allowing dry-leaf cannabis (the cheapest form) will have greater effects on outcomes. 4. *Opioid substitution provisions*: Laws with provisions allowing substitution of cannabis for opioid prescriptions will increase the pool of chronic pain patients eligible to use cannabis and have greater effects on outcomes relative to laws without such provisions. 5. *Opioid use disorder provisions*: Relative to laws without such provisions, laws that make opioid use disorder a qualifying condition will be associated with reduced use of non-cannabis treatment for opioid use disorder and increased treatment utilization for cannabis use disorder and poisoning, among patients with co-occurring chronic pain and opioid use disorder (**aim 2 only**). 6. *Registration fees*: Medical cannabis laws implemented without registration fees or with low-income subsidies will have greater effects on outcomes among patients with chronic non-cancer pain, which is disproportionately prevalent in low-income individuals. 7. *Dispensary limits*: Relative to states with no limits, states with dispensary limits will have lesser effects on outcomes. 8. *Local dispensary prohibitions*: Laws’ effects on outcomes will be stronger when localities allow dispensaries. 9. *Dispensary proximity*: Medical cannabis laws’ effects will be stronger among patients who live near a dispensary. 10. *Physician proximity*: Laws’ effects will be stronger among patients who live near a physician registered to recommend cannabis to patients.	

#### Aim 3

Study aim 3 is to characterize implementation of state medical cannabis laws for treatment of chronic non-cancer pain. Through interviews with state decision-makers and healthcare system leaders, we will collect in-depth information on leaders’ perceptions of Proctor’s eight implementation outcomes [32] as well as characterize key implementation strategies such as presence of state initiatives designed to support use of medical cannabis for treatment of chronic non-cancer pain and healthcare system policies related to medical cannabis treatment.

#### Aim 4

Study aim 4 is to characterize physician and patient perspectives of state medical cannabis laws as they pertain to the treatment of chronic non-cancer pain and to quantitatively measure four implementation outcomes: acceptability, appropriateness, adoption, and penetration. Through representative surveys of physicians and chronic pain patients in states with medical cannabis laws, we will examine perceived acceptability and appropriateness of medical cannabis for treatment of chronic non-cancer pain; the proportion of physicians who recommend medical cannabis to chronic non-cancer patients, refer patients to a recommending physician, or recommend *against* medical cannabis for chronic non-cancer pain management; and the proportion of people with chronic non-cancer pain who report using medical cannabis for pain management. Surveys will also measure barriers and facilitators to the use of cannabis for chronic non-cancer pain. Stratified analyses will explore whether relevant attitudes, for example patient perceptions of medical cannabis access, differ depending upon the policy implementation rules of the medical cannabis law in respondents’ state of practice (physicians) or residence (patients).

### Study design

The study uses a concurrent-embedded design [50], in which one primary method (difference-in-differences analyses, aims 1–2) guides the research, and secondary qualitative (aim 3) and survey (aim 4) methods play a supportive role. Aims 1–3 begin concurrently. Aim 3 interview results will inform aim 4 survey development; aim 4 will be conducted concurrently with the later phases of aims 1–2.

The study sample includes 32 states: 17 control states without medical cannabis laws (AL, GA, ID, IN, IA, KS, KY, MS, NE, NC, SC, SD, TN, TX, VA, WI, WY) and 15 intervention states that enacted medical cannabis laws in 2012 or later and do not have recreational cannabis laws (AR, CT, FL, LA, MD, MN, MO, NH, NY, ND, OH, OK, PA, UT, WV). We excluded states that enacted medical cannabis laws prior to 2012 due to concern about recall bias in aim 3 and excluded states that have enacted both medical and recreational cannabis laws since 2012 due to our study’s focus on medical cannabis laws; recreational cannabis laws could lead people to “self-medicate” with cannabis obtained through recreational channels and contaminate aim 1–2 quantitative analyses.

### Study period

The study period for the overarching study is 2009–2022, a period chosen to include 3 years of pre-law data for the states with the earliest (2012) medical cannabis law enactment dates in the sample. In quantitative aims 1–2, each of the 15 intervention states with medical cannabis laws will have a unique 7-year study period, with 3 years of data pre- and 4 years of data post-law. The rationale for this approach is that it is critical to examine the effects of state medical cannabis laws among continuous cohorts of patients with chronic non-cancer pain in order to attribute observed effects to medical cannabis laws as opposed to the changing composition of the study sample. But, requiring continuous presence of individuals in the aims 1–2, insurance claims data across the entire time period would substantially reduce sample size; we therefore will only require continuous enrollment for the 7-year study period relevant for each state. Aim 3 qualitative interviews will seek to characterize implementation timing, barriers, and strategies from the date a state’s law was enacted through the time interviews are conducted in 2021/2022. Aim 4 surveys will characterize physicians’ and patients’ attitudes and behaviors at the time the surveys are fielded in 2022.

### Data sources

#### Aim 1–2 state medical cannabis law data

Our study team assembled a longitudinal state medical cannabis law database using legal research and legislative history techniques, including full-text searches of the Westlaw database and identification of state session laws and regulatory materials. The longitudinal database includes each law’s effective date, date the first dispensary opened, and time-varying measures of the policy implementation rules of interest (see Table 1). For quality control purposes, we compared our findings with publicly available materials compiled by the Prescription Drug Abuse Policy System [35] and the National Conference on State Legislatures [34]. When we found inconsistences between our results and these materials, we consulted the text of the relevant law and sought clarification from legal experts in the relevant state.

#### Aim 1–2 administrative insurance claims data

Aims 1–2 will use Medicare and OptumLabs Data Warehouse administrative claims. The Medicare data includes inpatient, emergency department, outpatient, and prescription drug insurance claims for the approximately 34 million adults aged 65+ and nine million adults aged 18–64—who qualify for Medicare coverage by virtue of disability—covered by fee-for-service Medicare each year [51, 52]. The OptumLabs data used for this study include inpatient, emergency department, outpatient, and prescription drug insurance claims for approximately 30 million privately insured adults aged 18–64. Both data sources include data from all 50 US states. Information in these two claims data sources includes diagnosis codes; procedure codes; type, dose, and duration of prescriptions; and service dates, allowing for identification of individuals diagnosed with one of the five chronic non-cancer pain conditions of interest and the pharmacologic and non-pharmacologic pain treatments they receive. Unique patient identifiers allow tracking of individuals over time and across treatment settings. Patient demographic information includes age, sex, state, and five-digit zip-code of residence. Unique provider identifiers allow tracking of providers over time. Provider characteristics include specialty and treatment setting.

#### Aim 3 qualitative interview data

Aim 3 qualitative data will be collected through semi-structured interviews with key state policy and healthcare system leaders in the 15 intervention states. The guide will include three cross-cutting domains relevant for both groups of interviewees: (1) perceptions of policy implementation rules; (2) perceived barriers and facilitators to implementation of state medical cannabis laws for the treatment of chronic non-cancer pain; and (3) perceptions of Proctor’s eight implementation outcomes [32] in the context of medical cannabis law implementation for chronic non-cancer pain. The interview guide for state policy leaders will include two additional domains focused on (a) eliciting leaders’ perceptions of factors influencing the design of medical cannabis policy implementation rules and (b) characterizing state medical cannabis law implementation initiatives. The interview guide for state healthcare system leaders will include two domains in addition to the cross-cutting domains above, which will focus on (a) healthcare system leaders’ perceptions of how their state’s medical cannabis law has influenced treatment of chronic non-cancer pain and (b) characterizing healthcare system policies or other initiatives related to medical cannabis.

The interview guide will be developed by the study team and refined based on feedback from the study’s advisory board, which includes national and state experts in pain management, medical cannabis, addiction medicine, and drug policy. Videoconference interviews will be conducted by a single master’s-level study team member trained in qualitative interviewing techniques. Table 2 delineates our qualitative research design within the Consolidated Criteria for Reporting Qualitative Studies (COREQ) framework [53], including additional details regarding interview guide development and data collection.

**Table 2:** Qualitative study design

Research team and reflexivity	
Personal characteristics	
1. Interviewer/facilitator	All interviews will be conducted by the same member of the study team.
2. Credentials	The interviewer will be a masters-level trained research assistant.
3. Occupation	The interviewer will be employed full-time as a research assistant.
4. Gender	The interviewer will be female.
5. Experience and training	The interviewer will have experience participating in qualitative research studies and will be supervised by the study PI, who has extensive training and experience conducting qualitative research.
Relationship with participants	
6. Relationship established	Potential interviewees will be contacted with a standardized recruitment email to introduce the study and the interviewer and to request their participation.
7. Participant knowledge of the interviewer	The recruitment email will explain the study goals and why the interviewer is interested in conducting this research. This information will be reviewed at the start of each interview.
8. Interviewer characteristics	The recruitment email will provide information about the research team, including the interviewer. This information will be reviewed at the start of each interview.
**Study design**	
Theoretical Framework	
9. Methodological orientation and theory	The qualitative portion of the study will use a content analysis approach.
Participant Selection	
10. Sampling	Potential interviewees will be selected based on their legally established responsibilities relative to the state law(s) of interest.
11. Method of approach	Potential interviewees will be approached with a standardized recruitment email.
12. Sample size	We anticipate conducting 3–5 interviews in each of the 15 intervention states.
13. Non-participation	We will document any reasons provided by those who decline to participate as well as any individuals who do not respond to our recruitment email.
Setting	
14. Setting of data collection	Data will be collected via interviews conducted by telephone or videoconference.
15. Presence of non-participants	We anticipate that the interviewer and interviewee will be the only individuals present.
16. Description of sample	The sample will include key implementation leaders for the law(s) of interest in each of 15 intervention states.
Data collection	
17. Interview guide	The interview guide will be developed by the study team and shared with an advisory board for feedback. It will be pilot tested and refined before data collection begins.
18. Repeat interviews	We will conduct repeat member-checking interviews with a random sample of 20–30 interviewees.
19. Audio/visual recording	Once permission is granted, videoconference interviews will be recorded.
20. Field notes	The interviewer will draft summary notes immediately after concluding each interview.
21. Duration	We anticipate that interviews will last no more than 60 min.
22. Data saturation	The study team will convene on a regular basis to review interview data and determine when data saturation is reached.
23. Transcripts returned	We do not plan on returning transcripts to interviewees. Based on the straightforward nature of our questions and prior research with similar types of interviewees, we do not anticipate that this will be necessary.
**Analysis and findings**	
Data analysis	
24. Number of data coders	We plan to have two coders pilot a sub-sample of transcripts. Once discrepancies are resolved and the codebook is finalized, the full set of transcripts will be coded by one individual.
25. Description of the coding tree	We plan to develop a coding tree (i.e., codebook) based on a review of the literature, a priori knowledge within the study team, and summary notes from interviews. We will also share a draft codebook with our advisory board for feedback.
26. Derivation of themes	Themes will be derived once data have been coded. Preliminary themes may be identified based on discussions with the interviewer and review of field notes.
27. Software	We plan to use NVivo qualitative research software.
28. Participant checking	A bulleted list of key findings will be shared with participants once data have been coded and analyzed.
Reporting	
29. Quotations presented	Quotations from interviews will be used to present findings, and they will be accompanied by an interviewee identification number.
30. Data and findings consistent	Our planned use of quotations will allow for assessment of consistency between our data and findings. We will also create supplemental tables with additional quotations to share as much information as possible when presenting our findings.
31. Clarity of major themes	We plan to use sub-headings listing our major themes to promote clarity when writing up our findings.
32. Clarify of minor themes	We plan to provide quotations from interviewees who raised minor themes or shared information contrary to findings of our major themes.

#### Aim 4 survey data

Aim 4 data will be collected through surveys of representative samples of three groups: (1) all primary care and pain specialist physicians, (2) primary care and pain specialist physicians registered to recommend medical cannabis in their state, and (3) people with chronic non-cancer pain in the 15 intervention states. Physician surveys will be administered by the study team using a multi-contact, mixed-mode strategy following an adapted Dillman tailored design method [54], which includes three email survey waves and three postal survey waves. Patient surveys, which will be administered by NORC using their nationally representative AmeriSpeak panel, will be conducted by telephone or online, depending upon AmeriSpeak panel members’ preference [55]. Survey domains and item development will be informed by aim 3 interview results. See the measures section below for preliminary domains. Table 3 characterizes our survey research design within the Survey Reporting Guideline (SURGE) framework [56], including additional details regarding survey development and data collection.

**Table 3:** Survey research design

	Survey 1: All primary care and pain specialist physicians in intervention states	Survey 2: Primary care and pain specialist physicians registered to recommend cannabis to patients	Survey 3: People with chronic non-cancer pain
**Survey administration**			
33. Questionnaire administration	Mixed-mode email/postal survey		Online survey fielded by AmeriSpeak. Panel members can choose either web or telephone survey administration.
34. Dates of data collection	Anticipated January–June 2022		Anticipated May–June 2022
35. Number and types of contact	Up to 8 contacts: introductory letter delivered by email and post; email survey waves 1–3; postal survey waves 1–2; reminder postcard; postal survey wave 3. Once a physician responds to the survey, they will not receive further contact.		Up to 10 contacts; email or telephone follow-ups to non-responders depending on preferred mode of administration.
36. Data entry	Email survey responses will be captured directly in an electronic database. Postal survey responses will be double-entered into an electronic database by two research team members; discrepancies will be identified and reconciled.		Web survey responses will be captured directly in an electronic database. Telephone responses will be entered into an electronic database by AmeriSpeak staff.
**Sample selection**			
37. Sample frame	Primary care and pain specialist physicians in the National Provider Plan and Enumeration System (NPPES), a physician database maintained by the Centers for Medicare and Medicaid Services (CMS).	Primary care and pain specialist physicians registered to recommend medical cannabis in the 10 study states with medical cannabis laws that require physicians to register with the state in order to recommend cannabis to patients.	NORC’s AmeriSpeak Panel of ≈35,000 adults aged 18+.
38. Sample size calculation	Sample sizes were determined based on a margin of error calculation showing that with the expected number of completed surveys (*N* ≈ 1000 for all three surveys), results would have a margin of error of 2–3 percentage points.		
39. Representativeness	The NPPES sample frame includes all US physicians who bill government and commercial insurers.	The sample frame includes all physicians registered in their state to recommend cannabis.	The AmeriSpeak panel is sourced from NORC’s area probability sample and from a US Postal Service address-based sample covering 97% of US households.
40. Method of sample selection	Simple random sample		
41. Sample size	*N* = 2000 fielded surveys; expected response rate 50%, for *N* ≈ 1000 completed surveys.		*N* = 1500 fielded surveys; expected response rate 70%, for *N* ≈ 1000
**Survey instrument**			
42. Instrument development	The research team will develop domains and preliminary items based on the research questions and on the results of aim 3 qualitative interviews. We will use items with established reliability and validity when available, and conduct cognitive interviewing with convenience samples of physicians and patients to support development of new items.		
43. Pre-testing	The study team will pre-test each survey with ≈25 physicians		NORC will pre-test the survey with ≈25 AmeriSpeak panelists.
44. Reliability and validity	In addition to using established items when available and conducting cognitive interviewing as noted above, we will conduct exploratory factor analysis to assess reliability of items within domains.		
45. Scoring methods	We will examine the distribution of responses to each survey item using descriptive statistics.		
**Response rates**			
46. Response rate calculation	Response rate will be calculated as the proportion of eligible physicians who complete the survey.		Completion rate will be calculated as the proportion of AmeriSpeak panelists selected for the survey who complete the survey. Response rate will be calculated to incorporate the panel recruitment rate as well as the completion rate per AAPOR guidelines for probability-based panel surveys.
47. Nonresponse	For all surveys, we will assess whether measured characteristics differ among respondents versus non-respondents. Survey weights will adjust for differential response.		
**Reporting**			
48. Alignment with objectives	Results reporting will align with study aims.		
49. Sub-group results	Sub-group analyses will align with study aims and sub-group Ns will be reported.		

### Study sample

The aim 1–2 study sample will include individuals continuously enrolled in Medicare or private insurance for their state’s 7-year study period and individuals in control states who are continuously enrolled for the same 7-year periods as the intervention states. The sample includes people were diagnosed with a chronic non-cancer pain condition—low back pain, fibromyalgia, chronic headaches, arthritis, or neuropathic pain—during the study period.

The aim 3 study sample will include key state policy and healthcare system leaders in the 15 intervention states. We will begin by interviewing (1) the individual with primary responsibility, as determined by statute, for implementation of the state’s medical cannabis law, and (2) the Chief Medical Officers of two large integrated healthcare systems in each state (when more than two exist, we will select one in an urban area and one in a rural area, if possible). Additional interviewees will be identified through purposive snowball sampling [57]. Interviewees will receive a standard recruitment email explaining the study aims and inviting them to participate in the study. We plan to conduct interviews with 3–4 policy leaders and 3–4 healthcare system leaders in each intervention state.

In aim 4, we will survey three samples. First, we will survey a random sample of 2000 primary care physicians and 2000 pain specialist physicians practicing in the 15 intervention states. The sample will be drawn from the National Provider Plan and Provider Enumeration System (NPPES), a Centers for Medicare and Medicaid Services (CMS) physician database. Second, we will survey 2000 primary care physicians and 2000 pain specialist physicians registered to recommend medical cannabis in the 10 intervention states that require physicians to register with the state in order to recommend cannabis to patients. The sample will be drawn from state databases obtained by our study team. Third, we will survey a representative sample of 1500 people with chronic non-cancer pain in the 15 intervention states using the NORC AmeriSpeak panel [55], a nationally representative, probability-based survey panel. We will identify people with chronic non-cancer pain using the National Health Interview Survey metric on national prevalence [2].

### Measures

Aim 1–2 independent variables are dichotomous indicators of medical cannabis laws that will “turn on” (from 0 to 1) in the first full year the law is implemented. Aim 1–2 effect modifiers, or “moderators,” are dichotomous indicators of each of the specific policy implementation rules (Table 1), except dispensary and registered physician proximity. These variables will be measured as the distance in driving miles from the centroid of patients’ residential zip code to the nearest dispensary/registered physician.

Aim 1 dependent variables include measures of receipt of opioids, non-opioid pain medications, and non-pharmacologic therapies (e.g., physical therapy). Our study team identified non-opioid pain medications and non-pharmacologic therapies recommended by clinical guidelines to treat low back pain, headache, fibromyalgia, arthritis, and/or neuropathic pain. We will create person-year level measures indicating whether an individual received any opioid medication, any guideline-concordant non-opioid pain medication—with guideline-concordance defined as receipt of a medication recommended for that individual’s specific chronic non-cancer pain diagnosis—and any guideline-concordant non-pharmacologic therapy. Among individuals who received these treatments, we will measure the number of treatments per year. Aim 2 dependent variables include person-year measures of any inpatient, emergency department, or outpatient visits for opioid use disorder, opioid overdose, cannabis use disorder, and cannabis poisoning. Among individuals with any utilization, we will measure number of visits per person-year.

Aim 3 qualitative interviews will characterize the implementation of medical cannabis laws as they pertain to chronic non-cancer pain in the 15 intervention states. Key themes will be identified within the domains described in the data collection section above. These key themes will inform development of aim 4 survey domains by identifying issues related to medical cannabis law implementation that warrant further exploration through surveys of representative samples of physicians and patients. Preliminary aim 4 domains include:

*Survey 1, primary care and pain specialist physicians*: perceived acceptability and appropriateness of medical cannabis for chronic non-cancer pain; perceptions of demand for cannabis among chronic non-cancer pain patients; perceived effectiveness of cannabis as a treatment for chronic non-cancer pain; and self-reported chronic non-cancer pain treatment practices, including but not limited to recommendation of medical cannabis for management of chronic non-cancer pain (a measure of adoption). As this survey will be fielded among a representative sample of physicians, we will measure medical cannabis law penetration as the percent of all physicians who report recommending medical cannabis to patients with chronic non-cancer pain.

*Survey 2, physicians registered to recommend cannabis to patients*: measures of adoption including self-reported practices regarding recommending, referring, and monitoring cannabis for chronic non-cancer pain; perceived barriers and facilitators to delivering cannabis treatment; factors that influence treatment modality decisions, e.g., cannabis versus opioids versus non-opioid treatments.

*Survey 3, people with chronic non-cancer pain*: We will measure the policy implementation outcome measure of adoption as self-reported use of medical cannabis to manage chronic non-cancer pain. The survey will also measure self-reported use of prescription opioids, non-opioid prescription medications, and non-pharmacologic therapies to manage chronic non-cancer pain; perceptions of barriers and facilitators to cannabis treatment; and policy attitudes, for example whether or not insurers should cover medical cannabis.

### Analysis

#### Aim 1–2 difference-in-differences study

We will use a difference-in-differences design with a policy trial emulation approach to compare trends in outcomes pre/post medical cannabis laws in intervention states to changes in outcomes in control states over the same period. We will identify individual patients with chronic non-cancer pain for inclusion in the analytic comparison groups by adapting methods used in comparative effectiveness research to account for variation in law implementation date across states and taking advantage of longitudinal data on each person [58–61]. This method, as applied to public policy evaluations, has recently been described as “policy trial emulation” [49]. This approach is conceptualized as a series of all possible initiation trials over time, where each year a state implemented a medical cannabis law represents the start of a new trial, and comparison individuals are selected relative to that start year; data across trials are pooled.

As noted in the study sample section above, each intervention state has its own 7-year study period and sample of individuals continuously enrolled during that 7-year period. For each intervention state, we will create a control group using the year of law implementation to define the “time zero”: individuals in states without medical cannabis laws continuously enrolled during a given intervention state’s 7-year study period will be weighted by fitting a model predicting the probability of living in the medical cannabis law state as a function of observed patient characteristics, measured before the law’s implementation year. Individuals in intervention states receive a weight of 1 and comparison individuals receive a weight proportional to (1-*p*)/*p*, were *p* = probability of living in a medical cannabis law state. Comparison individuals in states with no medical cannabis laws can be used in multiple trials for intervention states implementing medical cannabis laws at different time points; variance estimation will account for those repeated observations and for the clustering of individuals over time and within states [62, 63].

We will use a flexible regression framework, fit in the weighted sample, to evaluate medical cannabis laws’ effects on outcomes. Consider *Y* any of the outcome measures, *f*(∙) a function to transform *Y* (e.g., logit), law an indicator for the intervention group, and Post an indicator of whether the observation is “post” medical cannabis law adoption. The basic difference-in-differences model is: (1) *f*(*Y*) = α + β1Law + γ2Post + *δ*law*post + ε. δ is the coefficient of interest: the effect of exposure to a state medical cannabis law on outcome *Y*. In addition to main models estimating the average effects of state medical cannabis laws over the entire post-law period, we will separately estimate effects for years 1–4 post-law by allowing *δ* to vary by year post-implementation. Effect modification will be measured with a three-way interaction between law, post, and the modifier (as well as all two-way interactions of those 3 variables). While aim 3–4 measures of policy implementation outcomes are primarily designed to support interpretation of aim 1–2 model results, we will explore the possibility of including state-level implementation outcome measures (e.g., the proportion of physicians in a given state in the aim 4 survey sample who report recommending cannabis for chronic non-cancer pain, a measure of penetration) as effect modifiers in aim 1–2 analyses.

#### Aim 3 qualitative analysis

After each interview, the interviewer will create a summary memo to help identify preliminary themes within the data. Interview transcripts will be analyzed using a staged approach, starting with general coding and then including more specific codes as data analysis proceeds and researchers develop and refine a working model for the relationships within the data. The study team will create an initial codebook based on the research questions and summary notes from interviews. The codebook will be refined through input from the study’s advisory board. Then, two coders will pilot the codebook using a randomly selected sub-sample of transcripts. Discrepancies between the two coders will be resolved through a discussion and consensus process with the full study team. If additional themes emerge during the pilot phase, they will be added to the codebook. In the case of significant disagreements, we will solicit additional review from the advisory board. The finalized codebook will be applied to all transcripts. Qualitative research software will be used to organize text segments, first descriptively and then by themes and sub-themes. See Table 2 for more details.

#### Aim 4 survey analysis

All analyses will incorporate sampling weights adjusting for known sampling deviations and survey nonresponse. We will calculate the proportion, with 95% confidence intervals, of respondents endorsing each survey item response option. Statistical significance of potential sub-group comparisons, for example planned comparisons of survey responses among respondents living in states with differing medical cannabis policy implementation rules, will be assessed using chi-square tests. See Table 3 for more details.

## Discussion

Triangulation of the results of an econometric policy evaluation, qualitative interviews with key state medical cannabis law implementation leaders, and surveys of physicians who treat and patients who experience chronic non-cancer pain strengthens our ability to make causal inferences about the effects of state medical cannabis laws on treatment of chronic non-cancer pain, opioid use disorder and overdose, and cannabis use disorder and poisoning. This study design overcomes methodological limitations of existing ecological studies using aggregate state-level data.

A primary contribution of our study is consideration of how medical cannabis policy implementation rules influence outcomes. Policy implementation is often a “black box” in quantitative policy evaluations, despite its considerable influence on whether and how a policy affects outcomes and high relevance to decision-makers. In the context of state medical cannabis laws, we currently operate in an environment where US states without medical cannabis laws are considering enacting such laws *and* decision-makers in the 33 US states and Washington, D.C., with medical cannabis laws are considering amendments to the rules of their existing laws. This study is designed to improve upon traditional quantitative state policy evaluations, which yield information about the average effects of a given type of state law across states with varying implementation rules, to produce actionable information about the degree to which specific policy implementation rules affect outcomes.

Most extant policy implementation research has focused on strategies for enacting evidence-based policy [19–26]. This study exemplifies one approach for studying the implementation of enacted public policies, using quantitative and qualitative measures of implementation outcomes, such as acceptability and adoption, to inform interpretation of a quantitative econometric study examining policy effects on patient outcomes. Triangulating policy implementation outcomes with findings regarding the policy’s effects on patient outcomes strengthens the ability to make accurate inferences about medical cannabis laws’ effects on outcomes among patients with chronic non-cancer pain. In the absence of robust implementation, medical cannabis laws are unlikely to drive significant changes in the treatment of chronic non-cancer pain. Our study has several limitations. The aim 1–2 administrative claims data do not capture recommended chronic non-cancer pain therapies not covered by insurance, like yoga. The claims-based measures cannot differentiate between the dose and types of cannabis used by patients with cannabis use disorder or poisoning, and the ICD codes used to identify cannabis poisoning do not distinguish between synthetic and natural cannabis. Some patients with chronic non-cancer pain transition from opioids prescribed by a physician to illicit opioids, but our data does not capture measures of illicit opioid use. The subgroup of individuals in the aim 4 survey who use medical cannabis to manage their chronic non-cancer pain is expected to be small, limiting our ability to characterize attitudes and reported practices in this group.

While some policymakers and advocates have promoted state medical cannabis laws as a solution to the opioid epidemic on the premise that individuals with chronic non-cancer pain may substitute cannabis in place of opioids for pain management [64, 65], rigorous evidence is lacking. Our study uses a mixed-methods approach to study whether and how implementation of medical cannabis laws influences receipt of opioid and non-opioid treatment, as well as adverse opioid- and cannabis-related outcomes, among patients with chronic non-cancer pain.

## Data Availability

Please contact the authors for data requests.
